# Lost in the Edit: Reclaiming the Clinical Narrative in the Age of Synthetic Records

**DOI:** 10.7759/cureus.100866

**Published:** 2026-01-05

**Authors:** Shaheen E Lakhan

**Affiliations:** 1 Bioscience, Boricua Bio, San Juan, PRI; 2 Bioscience, Global Neuroscience Initiative Foundation, Miami, USA; 3 Neurology, Western University of Health Sciences, Pomona, USA; 4 Neurology, A.T. Still University School of Osteopathic Medicine in Arizona, Mesa, USA; 5 Medicine, Morehouse School of Medicine, Atlanta, USA

**Keywords:** artificial intelligence in medicine, clinical documentation, clinical reasoning, electronic health records (ehr), healthcare quality and safety, medical education, narrative medicine, physician burnout

## Abstract

We are entering the era of the synthetic record, where ambient artificial intelligence (AI) and large language models have begun to automate the clinical encounter. While this technology promises to alleviate administrative burden, it brings a more profound risk: the erosion of explicit clinical reasoning and the human clinical narrative, with downstream consequences for patient safety, care transitions, reimbursement, auditing, and medicolegal accountability. This editorial examines the crisis of the clinical note through a cinematic lens, arguing that the modern record has evolved into a disjointed CGI blockbuster that prioritizes data-rich special effects over a coherent script. It identifies 10 distinct audiences, from patients and forensic auditors to data-mining algorithms, and demonstrates how attempts to satisfy these conflicting “studios” have fractured clinical reasoning. By introducing a taxonomy of narrative failures, including fluent but intent-poor AI-generated prose and unclosed diagnostic loops, this piece advocates for a shift toward omni-channel clinical production. In this paradigm, the clinician serves as the content originator of a single narrative synthesis that explains what was thought and why, while AI renders this core clinical intent into formats tailored for diverse stakeholders. Reclaiming the clinical note as a persuasive argument rather than a transactional receipt is not merely a professional courtesy; it is a safety imperative for the digital age.

## Editorial

Act 0: the narrative singularity

The conversation around clinical documentation has reached a critical inflection point. For a decade, the medical community has focused on the EHR burden - the clicks, the templates, and the data debris of a system designed for billers rather than healers. However, recently, a more existential threat has emerged: the narrative singularity. With the rapid adoption of ambient AI and generative models in the exam room [1], we have reached a point where the note can be written without the clinician ever touching a keyboard. The machine can now produce a syntactically perfect record of a conversation, but it remains fundamentally incapable of capturing clinical intent. We are no longer just fighting bloat; we are fighting the loss of the clinician’s voice. To save the clinical record, we must stop viewing documentation as a record of “what was said” and return to its true purpose: the story of “what was thought.”

Act I: the premise and the breaking point

Bad movies and bad clinical notes fail for the same reason. At a certain point, the audience can no longer follow or trust the story. Characters behave in ways that are unexplained, motivations are unclear, critical details are missing or appear out of sequence, and the viewer stops leaning in and starts questioning what they are seeing. In cinema, this loss of narrative coherence leads to disengagement and disbelief. In medicine, the consequences are far more concrete. Notes that fail to hold together invite denied claims, failed audits, unsafe handoffs, and legal vulnerability.

However, despite how often clinicians experience the downstream effects of poor documentation, the root cause is frequently misidentified. The failure is no longer one of volume or billing compliance. It is that the record no longer explains itself. Decisions appear without visible reasoning, conclusions arrive without paths, and the reader is left to reverse-engineer intent from fragments.

Act II: the fragility of modern clinical documentation

Clinical documentation has quietly become one of the most fragile points in modern healthcare. Despite unprecedented technological investment, clinical notes are now longer than ever yet often less meaningful, dense with data but thin on reasoning [2]. The prevailing critiques focus on usability burdens, copy-and-paste excesses, and the distortions introduced by reimbursement incentives. These critiques are not wrong, but they are incomplete. What has been lost is not merely efficiency or elegance, but coherence. Many modern notes technically contain all required elements and yet fail as clinical records because they do not explain why decisions were made. They record what happened without demonstrating how one conclusion led to another. When that narrative thread frays, the record becomes difficult to trust, even when it is factually accurate.

Importantly, these narrative failures long predate artificial intelligence (AI) and were present in paper-based and early electronic notes; AI does not create the problem, but risks amplifying it by accelerating documentation and increasing the likelihood that fluent text is accepted without verification of clinical intent.

This fragility is akin to a film that has been over-edited in post-production. Think of a big-budget blockbuster that feels hollow despite its visual splendor. The scenes are expensive, the actors are talented, but the thematic glue is missing. In the electronic health record (EHR), we have the CGI of automated laboratory imports and the special effects of high-resolution imaging, but the script - the clinician’s actual thought process - is often left on the cutting room floor. When we prioritize the data dump over the data synthesis, we are essentially providing the audience with a pile of raw footage and expecting them to edit the movie themselves.

Act III: a script for too many studios (the audience crisis)

A primary driver of this narrative collapse is the fact that the audience for a clinical note has undergone a radical transformation. Today, it is a public-facing legal and financial instrument being read by a dozen non-clinicians for reasons that have nothing to do with healing. The clinician-author is now forced to write one script that satisfies multiple studios with conflicting interests.

First, there is the patient, who is now the leading actor and a primary viewer. Under the 21st Century Cures Act, patients have immediate access to their notes via OpenNotes [3]. This requires a narrative shift; clinicians must balance technical accuracy with people-first language. Simultaneously, the clinician must write for the biller and coder, who act as script analysts looking for specific genre beats required for a certain rating. Close behind are the payer and the auditor, the critics who hunt for narrative plot holes - such as a lack of failed conservative therapy before an MRI - to justify a denial of payment.

Beyond the financial and legal realms, new invisible viewers have entered the theater. Quality and safety officers act as guild monitors, reviewing notes to ensure that mandated scenes are present to maintain the hospital’s national ratings. Utilization Management teams act as production managers, reviewing notes in real-time to decide if the character still belongs in the hospital. Finally, we have the researchers and data scientists - the film historians - who demand structured data and actively discourage the director’s cut because nuance and narrative ambiguity are a nightmare for data analysis.

Act IV: continuity errors and the mystery box of the EHR

In television production, a continuity error occurs when a glass of water is half-full in one shot and empty in the next. In the EHR, these errors are dangerous. We have all encountered the zombie note - a daily progress note copied forward for ten days. In this movie, the patient is perpetually resting comfortably, even as the vitals flow sheet shows them escalating to high-flow oxygen. Furthermore, many modern notes suffer from what filmmaker J.J. Abrams calls the "mystery box" - a narrative device where questions are raised but never answered [4]. A clinician documents a "suspicious mass on CT" in the assessment but never mentions it again. The reader is left hanging, wondering if the biopsy was ordered or if the clinician simply forgot. Every mystery box opened in a clinical note must eventually be closed, or the narrative integrity of the patient’s care vanishes.

Act V: a long view from medical education

Over a career spent teaching across the full clinical spectrum, one pattern is impossible to ignore. Earlier generations were taught to think and write in narratives. Notes explained findings and reasoning [5]. That discipline has eroded, accelerated by the shift toward templated completeness over coherence. In the classroom, we are witnessing the death of the clinical arc. Trainees are adept at finding data points, but they struggle to weave those points into a patient journey. If we do not teach clinicians how to build a clinical argument, we are failing to teach them how to think. Reasoning is a narrative process. To diagnose is to find the most likely story that explains the patient’s symptoms. When we decouple the note from that story, we strip the clinician of their most powerful diagnostic tool.

Act VI: the differential as alternate endings

In high-concept cinema, the ending is not a foregone conclusion. The audience is presented with alternate endings that keep the tension alive. Clinical reasoning follows this logic through the differential diagnosis. However, the modern templated note has killed the alternate ending. We see a diagnosis listed as a fait accompli, with no record of the competing theories discarded. A thought-leading approach treats the differential as the most critical part of the script - the director’s commentary on why certain plot lines were abandoned. Documenting why a patient does not have a pulmonary embolism is often more important for future safety than simply documenting why they do have bronchitis.

Act VII: the uncanny valley of ambient AI

The arrival of ambient AI mirrors the uncanny valley of CGI - the point where a digital character looks human but feels off. AI generates notes that are syntactically flawless but lack clinical intent. If we rely on AI to write the script, we risk creating polished fictions. An AI might bridge a narrative gap by hallucinating a connection - linking chest pain to anxiety because the patient mentioned a stressful move, while the clinician was actually worried about a pulmonary embolus. We must not mistake fluency for truth. The goal of documentation technology should be to amplify the clinician's voice, not to replace it with a generic script.

Many of these failures arise from longstanding EHR design and workload pressures rather than AI itself, but automation increases their scale and reduces the friction that previously forced clinicians to re-engage with the narrative.

Act VIII: the future of omni-channel production

We are entering an era of omni-channel clinical production. The clinician’s role shifts from a transcriptionist to a content originator. The clinician captures the raw footage of the encounter - the subtle nuances of the exam and the intuitive leap in the history. AI then acts as the post-production suite. Rather than producing one 20-page kitchen-sink note, the AI takes the clinician's core synthesis and renders it into multiple productions. The patient receives a clear narrative of their care. The biller receives a financial logic report. The quality officer receives a compliance log.

By reducing documentation friction, AI may paradoxically increase expectations for clinical throughput, potentially worsening cognitive load and burnout rather than alleviating it.

By decoupling intent from output, we allow the clinician to return to being a storyteller while satisfying the data-hunger of the modern system.

Act IX: the production design (a set not built for actors)

If we are directors, the EHR is our set. Most EHR interfaces are designed for the transaction, not the transformation. They prioritize the capture of discrete data elements - a term that sounds like a tax audit. When a clinician navigates thirty menus to describe a pain, the narrative flow is decapitated. This is a director restricted to pre-approved clips. To fix the note, we must rebuild the set. We need story-centered design where the narrative is the primary canvas, and data-capture is a silent assistant.

Act X: the sequel hook (narrative as strategy)

A stellar note anticipates the future through foreshadowing. In the ICU or ED, the note should contain contingency narratives. A clinician may write: "The current story is simple dehydration, but if the creatinine does not improve by morning, the script shifts toward obstructive uropathy." By explicitly stating pivot points, the clinician provides a roadmap for the next doctor. This transforms the note from a post-mortem of yesterday into a strategic script for tomorrow.

Act XI: the comprehensive taxonomy of narrative failure

The following framework categorizes common documentation failures not as simple clerical errors, but as fundamental breaks in the clinical argument (Table 1). Each dissonance represents a conflict between the clinician’s intent and the audience's interpretation.

**Table 1:** A taxonomy of narrative failures in clinical documentation The table categorizes common failures in clinical documentation not as clerical or formatting errors, but as disruptions of the underlying clinical narrative. Each category represents a mismatch between the clinician’s intent and how the record is interpreted by its diverse audiences. Framed through cinematic analogies, these narrative dissonances illustrate how loss of coherence, continuity, or explanatory reasoning can compromise patient safety, clinical communication, reimbursement, and legal defensibility in the era of electronic health records and AI-generated documentation.

Narrative Dissonance	The Cinematic Parallel	The Clinical Plot Hole	Primary Audience Conflict	Consequence to the "Production"
Diagnostic Inertia	The Zombie Sequel	Copying forward an old exam or assessment regardless of the patient's changing status.	Legal team vs. treating team	Proof of inattentional blindness: the script contradicts the reality of the scene.
Logic Gaps	The Plot Hole	Ordering high-risk therapy (e.g., anticoagulation) without documenting the underlying suspicion.	Auditors vs. clinicians	"Medical necessity" denied: the logic of the intervention is invisible.
Narrative Abandonment	The MacGuffin	Mentioning a major finding (e.g., lung nodule) once, then never closing the loop in future notes.	Patient vs. risk management	The "Incidentaloma" trap: a story thread left dangling that leads to litigation.
Polyphonic Discord	Unreliable Narrator	The Primary Clinician, Nurse, and Consultant notes provide three different versions of the patient’s "stability."	Interdisciplinary team	Breakdown of trust: "plot" confusion that leads to medical errors.
The Fluency Trap	The Uncanny Valley	AI-generated prose that sounds professional but hallucinates clinical connections.	The AI vs. the clinician	"Polished Fictions" that obscure true clinical intent with generic filler.
Metric Fixation	Script Doctoring	Prioritizing quality check-boxes (e.g., BMI counseling) over the primary life-threatening plot.	Quality officers vs. the patient	A "technically perfect" note that misses the human tragedy of the encounter.
Premature Closure	Deus Ex Machina	Removing a diagnosis from the problem list without documenting the negative workup.	Fellow clinicians	Forced "re-work"; the next doctor must re-diagnose what was already solved.
The "Kitchen Sink" Edit	The Over-Edited Trailer	A 20-page note of raw data that contains everything but says nothing.	Researchers vs. readers	"Information Hiding": the clinical reasoning is buried under a mountain of metadata.
Functional Silence	Biographical Void	Recording vitals and labs but failing to document the patient's ability to walk or eat.	Disability reviewers	Loss of benefits for the patient: the "character" has no life outside the hospital bed.
Semantic Alienation	Fourth Wall Break	Using jargon or stigmatizing labels that the patient reads in real-time.	The patient (OpenNotes)	Destruction of the therapeutic alliance: the patient feels like a "specimen."

Act XII: reclaiming the narrative and the director’s cut

We must stop viewing the note as a passive byproduct and start viewing it as a clinical argument. The director’s cut is the version that reflects the creator's vision. We need a clinician’s cut that prioritizes reasoning over reimbursement. A well-told story is, by definition, a well-documented case.

We must reintegrate narrative competence into training, moving away from data-first presentations. We must also demand EHRs that support synthesis. Furthermore, we must address the audience burden. If the note serves as both a clinical tool and a billing document, technology must allow for layering. A clinician should write a core narrative of care, while background metadata, such as structured fields required for billing, quality reporting, and secondary data use, can be captured or appended in parallel.

Conclusion: the final scene

The crisis of the note is a crisis of meaning. When we lose the story, we lose the patient. We have spent two decades optimizing for data capture, but we have sacrificed the explanatory power that makes medicine a human endeavor. A clinical note is not a receipt; it is a professional hand-off, a legal defense, and a pedagogical tool. Restoring narrative coherence is a safety imperative. We must treat the Assessment and Plan as a persuasive argument. When we reclaim the narrative, we ensure the story of care remains clear, continuous, and safe. The edit has gone on long enough; it is time for the clinician to step back into the director’s chair and tell the story of medicine as it was meant to be told.
